# HIV/AIDS, more than 25 years later: which challenges remain?

**DOI:** 10.1186/1753-6561-5-S1-L1

**Published:** 2011-01-10

**Authors:** Françoise Barré-Sinoussi

**Affiliations:** 1 Institut Pasteur, Unit of Regulation of Retroviral Infections, Department of Virology, 75724 Paris Cedex 15, France

## 

The discovery of HIV in 1983 originated from a collective adventure, which mobilized clinicians, researchers and patients altogether. This collaboration was crucial to rapidly expand the knowledge of the virus and develop the first diagnostic tests and antiretroviral therapy (ART).

More than 25 years after the discovery of the etiological agent responsible for AIDS, research priorities still remain care, treatment and prevention with the major objective of developing a preventive vaccine.

Today, we have gained significant insight into the virus pathogenesis. The evolution and progression of the disease caused by HIV is closely linked to a number of determinants of the virus itself and the host. We also know today that, very early after exposure to the virus, a massive depletion of CCR5+ CD4+ T memory cells associated with microbial translocation occurs in the gastrointestinal tract of HIV infected patients. HIV infection is clearly inducing an inflammation and a generalized and persistent T cell activation, which may play a role in the persistence of HIV infection, resulting from the establishment of permanent reservoirs into host cells and in different host compartments. The reduction of the size of these reservoirs is representing one of the main challenges for the development of future therapeutic strategies.

Among other challenges in therapy, we also need to better understand the mechanisms leading to the severe complications (cardiovascular diseases, accelerated aging, cancer...) observed in some patients on long-term ART. Again, whether the inflammatory response is contributing or not to these complications remains an opened question.

The early acute phase of HIV infection appears therefore to be crucial in determining disease progression. Given the importance of the innate immune responses in this very early phase following infection and in driving adaptive immunity, further research on innate immunity in HIV infection are certainly among priorities for elaborating future therapeutic and vaccine strategies.

New technologies are today available to address all these scientific challenges. But they will only be overcome with a multidisciplinary and translational research for the global benefit of humanity.

